# Cutaneous shedding in amphibians causes shifts in bacterial microbiomes

**DOI:** 10.1111/1749-4877.12858

**Published:** 2024-06-19

**Authors:** Chava L. WEITZMAN, Gregory P. BROWN, Karen GIBB, Keith CHRISTIAN

**Affiliations:** ^1^ Research Institute for the Environment and Livelihoods Charles Darwin University Casuarina Northern Territory Australia; ^2^ School of Natural Sciences Macquarie University Sydney New South Wales Australia

**Keywords:** *Rhinella marina*, shedding, skin, sloughing, symbiotic microbiome

## Abstract

Considerable research has focused on microbes on amphibian skin, as they act as the first line of defense against invading pathogens. This effort has generated substantial data on patterns across species, space, time, and ontogeny, alongside a growing list of beneficial antifungal symbionts. Though there is evidence of stability in amphibian skin microbial communities, there is also an indication that regular skin shedding reduces cultivable bacteria, with regrowth and recolonization in the period between sheds. This suggests that skin communities are in constant flux, and we lack an understanding of how the membership and structure of those communities are affected by shedding events. In this study, we conducted experiments on cane toads (*Rhinella marina*) to investigate the influence of shedding on skin microbiomes. We first used quantitative PCR to verify a positive correlation between bacterial loads and time in the days after shedding. We then resampled individuals over time to describe changes in community composition in the 38 h after shedding using amplicon sequencing. Similar to trends of bacterial loads, we found increases in alpha diversity over time after shedding, suggesting that shedding reduces bacterial diversity as it knocks down bacterial loads. During the 38‐h period, community structure became similar to pre‐shed communities in some individuals, but there was no consistent pattern in structural changes among individuals. In light of the amphibian chytridiomycosis pandemic, understanding how physiological events such as skin shedding affect beneficial bacteria and communities on amphibians would provide important insight into amphibian ecology.

## Graphical abstract

Trends of bacterial load and diversity on cane toad skin suggest that regular shedding events reduce bacterial abundance, consequently also reducing richness and causing a shift in community structure. After reductions due to shedding, bacteria regrow and recolonize until the next shed days later, suggesting that skin communities are constantly in flux, despite indications of stability at longer time frames.

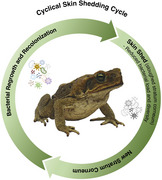

## INTRODUCTION

Animal skin has many functions, such as protection from the elements and playing a role in thermal balance, for example, through heat transfer in ectotherms or sweating in humans, alongside acting as a large sensory organ. Among tetrapods, amphibian skin plays an especially important role in homeostasis, participating in electrolyte and water balance, and acting as a site for respiration (reviewed in Voyles *et al.* 2011). Additionally, the skin is a site where bacterial symbionts expand the skin's protective role, acting as a first line of defense against invading pathogens and parasites. Though many frogs synthesize antimicrobial peptides in their skin, symbiotic skin bacteria may play a large role in innate immunity by providing what may be the main source of these peptides (Conlon 2011). It has also recently been proposed that skin bacteria interact with their host frogs in a manner consistent with an adaptive immune function, a role also acknowledged in other host taxa including humans (Harris‐Tryon & Grice 2022; Woodhams *et al.* 2023). As in other taxa, however, the outermost layer of amphibian skin periodically sheds, resulting in an environment that is constantly in flux, and the effects of these constant changes on the suite of physiological roles the skin plays in wild systems is still little understood.

Unlike many animals that shed their skin continuously (e.g. humans) or periodically in pieces (e.g. lizards), anurans (frogs including toads) shed skin from their whole body relatively frequently. For example, the frequency of skin shedding is on the order of months for snakes (up to 12 months in some taxa) (Stabler 1939), weeks to months for geckos (Fushida *et al.* 2020), and days to weeks for anurans. Tree frogs in the genus *Phyllomedusa* (Hylidae) can shed every day (Castanho & de Luca 2001), while some toads (Bufonidae) shed approximately every 10 days (Ohmer *et al.* 2019). Shedding frequency, however, can vary based on factors such as temperature and infection status (Meyer *et al.* 2012; Cramp *et al.* 2014; Ohmer *et al.* 2015). This process of shedding impacts the skin's capacity to perform functions as normal, as it results in decreased skin resistance and increased cutaneous water loss rates in a dry environment (Wu *et al.* 2017; Russo *et al.* 2018). However, these are paired with increased expression of aquaporin and junction genes, which help regulate the movement of water across the skin, and an increase in ion transport proteins, which together allow the animal to maintain its internal homeostasis (Wu *et al.* 2017, 2019). Alongside impacts on the skin's physiological functioning, we would expect skin shedding to impact bacterial communities with regular perturbations caused by frequent sloughing of a main site of bacterial symbioses.

Others have assessed the interconnectedness between frog shedding and the abundance of culturable bacteria, with increases in bacterial abundance between shedding events in two anuran species studied, Australian green tree frogs (*Litoria caerulea*) and cane toads (*Rhinella marina*) (Meyer *et al.* 2012; Cramp *et al.* 2014). While shedding can decrease bacterial loads, it can also decrease pathogenic fungal loads, at times leading to clearance of infection (Ohmer *et al.* 2017). This cyclical process of bacterial growth after being knocked down by shedding has thus far been studied using culturing to quantify bacteria, which considerably limits bacterial recovery and may not represent entire communities. Furthermore, how shedding impacts the bacterial community membership and structure is still unknown.

In this study, we used cane toads to assess shifts in skin bacteria after shedding, investigating changes in bacterial loads and identifying changes in community composition. We chose cane toads to address this topic because of the ease of maintaining individuals in captivity, established husbandry and sampling methods, and previous data collected on shedding (Meyer *et al.* 2012; Ohmer *et al.* 2019) and skin bacterial communities (Christian *et al.* 2018) existing for this species. Toads in this study were part of cohorts bred for multiple studies, and some individuals in the present study were included in another, larger experiment regarding infection in this species.

Through three experiments, we test the hypotheses: (1) that bacterial load increases with increasing time since shedding, supporting previous evidence that shedding reduces skin bacterial abundance; and (2) that shedding reduces richness (for instance, relatively rare taxa may disappear locally after shedding), and that community composition consequently shifts due to shedding as well. To address the first hypothesis, we first assessed whether there was a relationship between time since shedding and bacterial load and then assessed the nature of that relationship with increased precision. To address the second hypothesis, we used amplicon sequencing to evaluate the effects of shedding on skin microbial diversity in toads repeatedly sampled across time after shedding.

## MATERIALS AND METHODS

To test for the effect of shedding on toad skin bacterial loads, we did two experiments. In the first experiment, we used a coarse measurement of time between shedding and swabbing to determine if there was a relationship between time and whole‐body bacterial loads. In the second experiment with a larger sample size and larger toads, we improved the precision in the measurement of time since shedding (from a coarse measurement of days to finer‐scale minutes since shedding) and removed the impact of variation in toad size by sampling within a fixed area of skin of adult toads. In a third experiment, we incorporated serial sampling of individual toads, in contrast with the single‐sampling regime of the first two experiments, to detect changes in bacterial community diversity over time since shedding. Experimental procedures were approved by the Animal Ethics Committees of Charles Darwin University (permit A21010) and Macquarie University (permit 2021/01).

### Experiment 1—Does shedding impact skin bacterial loads?

We assessed relative quantities of bacteria on 16 individual toads within 5 days since they last shed to test for a relationship between bacterial load and time since shedding. Toads were full siblings, hatched in March 2022 at Macquarie University's Middle Point Station (Northern Territory, Australia) from locally sourced parental toads. Tadpoles and metamorphosed toadlets were raised in 700 L tanks outdoors, with toadlets kept on air‐dried soil from Middle Point with available water, uniquely marking each toad with toe‐clipping. In early May 2022, we applied a Liquid Paper^TM^ (Newell Australia) paint dot on the top of each toad's head while still co‐housed. Once per day in the evening, we identified which toads had shed that date based on their toe‐clipping and reapplied the paint dot. These toads were part of a separate study and were consequently co‐housed until 2 days before swabbing when they were placed into single‐housed containers on air‐dried Middle Point soil with a water dish. At the time of sample collection, the toads averaged 43.8 ± 4.5 (SD) mm in length (snout–urostyle length, SUL) and 9.0 ± 2.4 g, and they were too small to sex.

In early May, we collected skin swab samples as previously described (Christian *et al.* 2018). Toads were rinsed with 75 mL ultra‐pure water and swabbed with a flocked swab (Copan FLOQSwabs, Copan Diagnostics Inc., Murrieta, CA) using 30 swab strokes to sample the entire body surface, avoiding the face and cloaca. Swabs were preserved in 300 µL Zymo DNA/RNA Shield (Zymo Research, Irvine, CA) and stored at –20°C until DNA extraction (see below).

### Experiment 2—What is the pattern of bacterial regrowth after shedding?

We further assessed the effect of time since skin shedding on skin bacterial loads to better determine the nature of the relationship, measuring the regrowth of skin bacteria on 40 adult toads (103 ± 7.5 mm SUL). Here, we increased the precision of measurement between the times of shedding and swabbing, while also removing the effect of variable toad sizes. Toads were from six different families that had been bred in captivity and reared in semi‐natural enclosures at Middle Point for 2 years. In October 2022, toads were individually housed in transparent plastic cages on a small amount of soil with a water dish. Cages were held in a shed with exposure to ambient environmental temperature and humidity at Middle Point. To estimate the length of time between shedding and sampling of the skin bacteria, we monitored the Liquid Paper paint dot presence on the top of each toad's head with mounted cameras above the cages, collecting an image every 10 min to approximate the time the toad shed (i.e. when the paint dot disappeared). Paint marks were reapplied to shed toads each evening. To maximize our sampling with available cameras, we sampled two rounds of 20 toads, with toads housed in the setup for 12 or 6 days. All sampled toads shed at least once while in the enclosures. The time of shedding was estimated using the time of the last image with the white marking just before shedding.

Before collecting skin bacteria with swab samples, we rinsed toads with 150 mL of ultrapure water to remove excess dirt and debris. With a single flocked swab per toad, we collected bacteria within a 31 mm × 31 mm square stencil area, avoiding touching the stencil with the swab, on both the dorsal (posterior left) and ventral (posterior right) sides of the toad. Swabs were stored in clean 1.5 mL microcentrifuge tubes at –80°C until DNA extraction. The time of swabbing was recorded to the nearest 5 min to calculate the time between shedding and sample collection.

### Experiment 3—How does periodic shedding affect microbiome diversity?

For seven additional adult toads, we collected a time series of samples (5–6 swabs per toad) to detect changes in individuals’ bacterial communities over time since shedding using the same fine‐scaled approach as in Experiment 2. Large adult female toads (115 ± 8.5 mm SUL; sourced and held as in Experiment 2) were used for this experiment, dividing the dorsal and ventral surfaces into a grid of six 31 mm × 31 mm squares (see Fig. 2a for sampling regime). Toads were rinsed and swabbed before being placed in a clean container with a Liquid Paper mark and a mounted camera to detect the time of shedding. Initial swabs were collected 11–204 h before shedding, and post‐shedding swabs were collected periodically over 38 h after shedding. Toads in this experiment were kept in a lab environment at room temperature.

### DNA extraction and analysis of relative bacterial loads

Bacterial DNA was extracted from swab samples with Norgen's Microbiome DNA Isolation Kit (Norgen Biotek Corp., Thorold, ON, Canada) using the protocol for preserved samples for Experiment 1 and the general protocol for the others. For each experiment, we used qPCR to calculate relative bacterial loads with the Earth Microbiome Project 16S rRNA gene 515F/806R primers (Caporaso *et al.* 2011; Apprill *et al.* 2015; Parada *et al.* 2016), including a 10‐fold serial dilution of a toad colon bacterial sample run in triplicate to produce a standard curve.

Data were analyzed using R version 4.2.1 in RStudio 2022.07.1 (RStudio Team 2020; R Core Team 2022). Within each experiment, we used linear models to test the prediction that the relative load of bacteria (log_10_‐transformed) in samples is affected by the length of time since the last shed. Where relevant, comparisons of model fit were based on AICc scores.

For Experiment 1, we modeled the relative load of bacteria in the samples predicted by the interaction between the time since the last shed (days) and toad size, with mass proving to be a better predictor than toad length. Bacterial loads in Experiment 2 toads were analyzed with the time since the last shed (days) and toad sex as predictors. We also investigated the effects of toad origin and swabbing group on bacterial load, but they were not significant and removed from the final model. Based on the shape of the curve, we compared models using the untransformed time values and log_10_(time since shed) and found that the log‐transformed predictor variable provided a better fit.

### Characterizing post‐shed communities

DNA from the third experiment repeated samples was used to characterize changes in the skin community through its re‐growth after shedding. Changes in community diversity over time were quantified with amplicon sequencing. Library preparation and sequencing were conducted at the Ramaciotti Centre for Genomics at the University of New South Wales using the Earth Microbiome Project 515F/806R primers (Caporaso *et al.* 2011; Apprill *et al.* 2015; Parada *et al.* 2016) to amplify the V4 section of bacterial 16S rRNA gene. Amplicons were sequenced on a MiSeq v2 with 2 × 250 bp paired‐end sequencing in a run with other toad samples.

Demultiplexed sequences were trimmed (forward: 240 bases, reverse: 230 bases) and denoised with dada2 in QIIME2 2022.8 (Callahan *et al.* 2016; Bolyen *et al.* 2019) with a maximum of 5 expected errors in the reverse reads and otherwise default parameters. The taxonomy of reads was classified with the Silva v138 515F/805R database (Quast *et al.* 2012; Yilmaz *et al.* 2014), and we removed all non‐bacterial (including mitochondrial and chloroplast) reads. To reduce the likelihood of spurious taxa, we further removed amplicon sequence variants (ASVs) that never exceeded 0.1% relative abundance in a sample and ASVs only found in a single sample. After inspecting rarefaction curves, we rarefied each sample's reads to the lowest depth (22 183 reads per sample) for diversity analyses. We used QIIME2 to extract alpha (richness, Shannon's entropy, Pielou's evenness) and beta (Jaccard, Bray–Curtis) diversity values.

We focused analyses on separately assessing the impacts of post‐shedding sample number (with 1 representing the pre‐shed sample and 2+ representing sequential post‐shed samples) and time since shedding on bacterial diversity metrics. We used linear mixed‐effects models in the lme4 package with toad ID as a random variable (Bates *et al.* 2015) to analyze the three alpha diversity metrics. Beta diversity metrics were analyzed using a block design with the adonis2 function in the vegan package (Oksanen *et al.* 2020), permuting within each toad individual.

Last, we calculated the variability for each toad, defined here as the average Bray–Curtis distance between samples (including all samples per toad, and separately for the post‐shedding samples). To remove the impact of low‐abundance ASVs, we further calculated variability when only abundant community members were retained (removing ASVs with maximum relative abundance <2%, rarefying to lowest read depth of 15 494 reads per sample). We used these data to detect the extent that community stability (or instability) was driven by shedding and shedding of rare taxa, with paired *t*‐tests.

Amplicon sequence data have been submitted to NCBI's Sequence Read Archive (BioProject accession ID: PRJNA1045825).

## RESULTS

### Bacteria regrows after skin shedding

**Figure 1:**
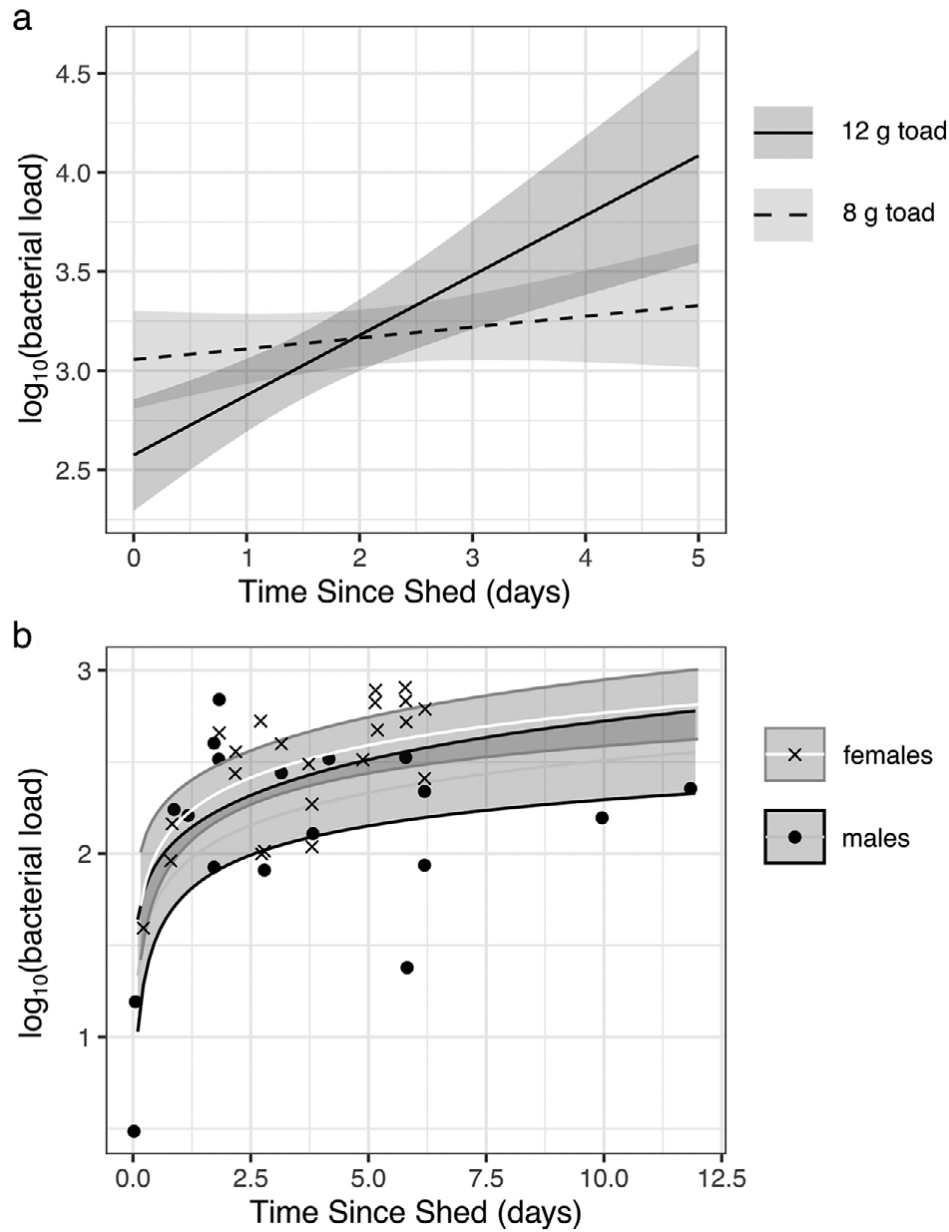
Bacterial load (values relative within each experiment) on cane toad skin increases with increasing time since shedding. (a) Fitted curves approximating changes in total‐body bacterial load on two sizes of subadult toads show a stronger increase over time on larger toads. (b) Fitted curves and raw values of bacterial loads on a standardized area of toad skin by toad sex show that females have higher loads than males.

Through two experiments, we found that shedding reduces bacterial loads on cane toads, with bacterial regrowth over time in between shedding events (Fig. 1). In the first experiment, days since the last shed (collected as whole integer days) significantly predicted the relative bacterial load in swab samples (model multiple *R* ^2^ = 0.65; days: *F*_1,12_ = 15.79, *P* = 0.002; mass: *F*_1,12_ = 14.26, *P* = 0.003; interaction: *F*_1,12_ = 20.40, *P* = 0.0007). From the significant interaction between mass and time, we found that larger toads experienced steeper increases in bacteria over time (Fig. 1a), likely as a consequence of the scaling of bacterial growth over larger skin surface areas. Of the 16 toads in this experiment, 10 shed twice between the time they were marked and the date they were swabbed, with an average intershed interval of 4.2 ± 0.63 (SD) days.

In the second experiment, we removed the influence of toad size by swabbing within a standardized stencil, and we increased the precision of our measurement of the time between shedding and sampling. The fitted curve and raw values in Fig. 1b represent bacterial loads on a standardized area of toad skin.

The results of this experiment revealed an effect of time since the last shed on a logarithmic scale (*F*_1,37_ = 34.26, *P* < 0.0001). We also found a difference between males and females (*F*_1,37_ = 5.52, *P* = 0.02), with female toads on average having 1.8× the bacteria of male toads. Ten toads shed twice before swabbing, with an average intershed interval of 6.3 ± 0.88 (SD) days.

### Shedding reduces bacterial community diversity

**Figure 2:**
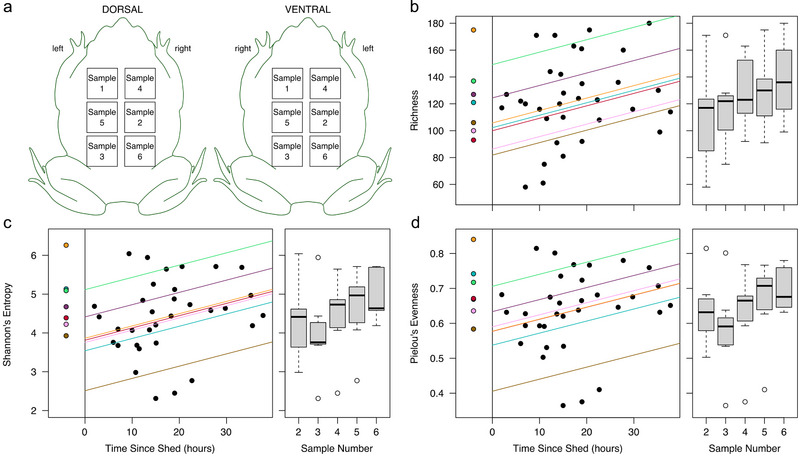
Microbial diversity was measured over time with repeated sampling of individual toads. (a) Sampling scheme, illustrating the sequential order of swabs collected for each toad. Sample 1 was collected before the toad shed, and samples 2–6 were collected during the first 38 h after the toad shed. Alpha diversity metrics in these samples increased with time after shedding. Each of (b) amplicon sequence variant (ASV) richness, (c) Shannon's entropy, and (d) Pielou's evenness were correlated with time since shedding (scatterplots) and post‐shed sample number (boxplots). Points on the far left of each scatterplot are pre‐shed values, for visualization purposes. Individual toads’ trend lines are color‐coded, corresponding with pre‐shed point colors.

We resampled individual toads over multiple body locations (Fig. 2a) to describe changes in bacterial diversity over time since shedding. Although not the focus of this third experiment, we measured bacterial load, but it did not correlate with time in post‐shed samples (linear mixed‐effects model, *χ* ^2^ = 0.086, df = 1, *P* = 0.8). This was unsurprising due to our sampling regime and expected heterogeneity of bacterial loads on different parts of toad skin, along with a cooler environment where the toads were housed, possibly impacting bacterial growth rates.

Sequencing of 16S rRNA gene amplicons from the third experiment resulted in 462 ASVs in 1 618 922 reads (average 40 473 reads per sample) after non‐bacterial and rare, potentially spurious, taxa were removed. All three alpha diversity metrics in post‐shed samples were significantly impacted by sample number (consecutive samples collected per toad) and time since shedding, with results showing a gradual increase in these metrics as time progressed since the animal last shed (Table 1 and Fig. 2).

**Table 1:** Statistical results of diversity analyses of bacterial community data in post‐shed samples from Experiment 3

	Sample number		Time since shedding	
Diversity metric	Test statistics	*P*	Test statistics	*P*
Richness	$X^{2}$ = 7.62, df = 1	*P* = **0.006**	$X^{2}$ = 5.24, df = 1	*P* = **0.02**
Shannon's entropy	$X^{2}$ = 7.76, df = 1	*P* = **0.005**	$X^{2}$ = 8.83, df = 1	*P* = **0.003**
Pielou's evenness	$X^{2}$ = 5.24, df = 1	*P* = **0.02**	$X^{2}$ = 7.08, df = 1	*P* = **0.008**
Jaccard	*F*_1,31_ = 0.69, *R* ^2^ = 0.022	*P* = **0.007**	*F*_1,31_ = 1.23	*P* = 0.5
Bray–Curtis	*F*_1,31_ = 0.92, *R* ^2^ = 0.029	*P* = **0.007**	*F*_1,31_ = 1.16	*P* = 0.6

Seven toads were repeatedly swabbed within a 31 mm × 31 mm stencil at various times after they shed. Each bacterial diversity metric from the swabs was separately analyzed against post‐shedding sample number (consecutive swab samples) and time since shedding. Richness, Shannon's entropy, and Pielou's evenness were analyzed with linear mixed‐effects models. Jaccard and Bray–Curtis metrics were analyzed with permutational MANOVAs. Bold denotes a significant predictor.

**Figure 3:**
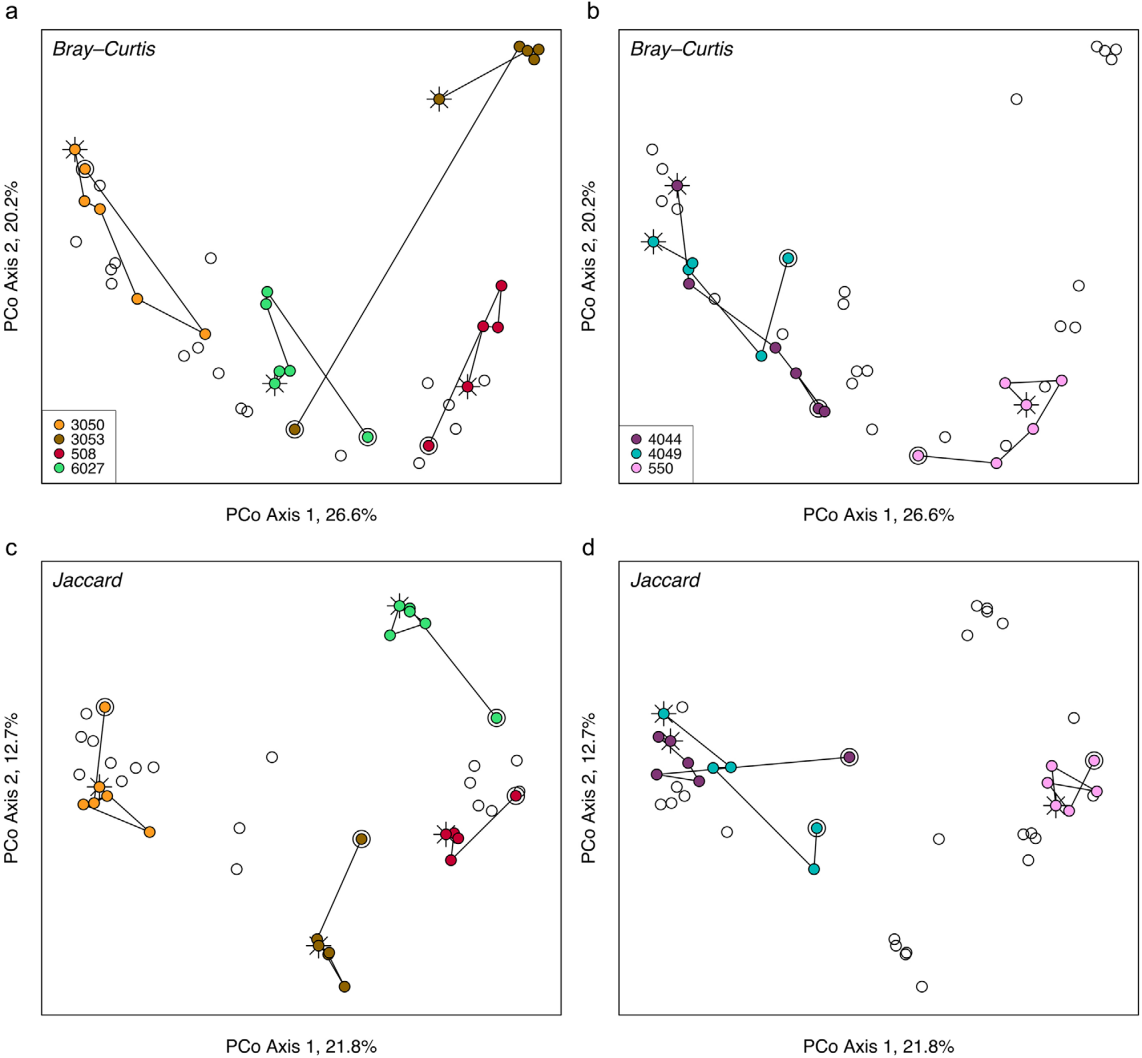
Beta diversity shifted from pre‐ to post‐shed communities, displayed in principal coordinates analyses, with Bray–Curtis dissimilarity illustrated in (a) and (b) and Jaccard distance illustrated in (c) and (d). Seven toads are divided into two panels for each diversity metric to best visualize the compositional shift after shedding in four toads (a,c), which was not observed in the remaining three toads (b,d). In each panel, points representing focal toads are color‐coded by toad ID, with open circles representing the remaining toads’ samples. Lines connect communities within a toad over time, from pre‐shed samples (bullseyes) to the final sample per toad (sunbeams).

Beta diversity metrics were influenced by sample number but not by time since shedding (Table 1). Though significant, sample number accounted for a small proportion of variation in the samples, with clear trends of post‐shed communities in only a few toads (Fig. 3). Visualizing Bray–Curtis dissimilarity (a measurement incorporating both presence/absence and relative abundances of community members) in an ordination revealed that communities in at least half of the toads became progressively more similar to pre‐shed compositions, despite a sharp contrast between pre‐ samples (sample 1 per toad) and those collected soon after the toad shed (Fig. 3a). This contrasts with Jaccard distance (based on presence and absence of taxa but not their relative abundances), which showed a difference between pre‐ and post‐shed community membership in most toads (Fig. 3c,d) with no shifts back toward pre‐shed compositions.

Variability (average Bray–Curtis distance) per toad was significantly greater by approximately 33% (*t* = 5.96, df = 6, *P* = 0.001) when pre‐shed samples were included in the calculation, signifying a strong influence of shedding on changes in community composition. Removing rare taxa decreased variability in the toads, but the effect of pre‐shed samples was still similarly high (34% greater variability when pre‐shed samples were included; *t* = 5.17, df = 6, *P* = 0.002). This suggests that shedding did not just impact the rare community members but more abundant members as well.

## DISCUSSION

Bacterial communities in and on wildlife are constantly impacted by environmental perturbations, disruptions by disease and host physiological stressors, and losses through regular host physiological processes such as tissue sloughing (Cramp *et al.* 2014; Jiménez & Sommer 2017; Greenspan *et al.* 2020). We investigated the impacts of cane toad skin shedding on skin bacterial load and structure and found support for our hypotheses, suggesting that shedding regularly disrupts skin communities by reducing not just their overall abundance, but also richness.

Our results confirmed previous findings of increasing bacterial loads between shedding events on anurans (Meyer *et al.* 2012; Cramp *et al.* 2014). Our two experiments investigating this trend differed in the relationship between time and bacterial load, probably due to the low‐precision measurement of time since shedding in the first experiment (whole days). Additionally, toads in the first experiment were co‐housed until 2.5 days before swabbing, allowing for greater reinoculation for those that shed before being single‐housed, impacting the shape of the correlation.

While toads were being held during the days preceding sampling for bacterial load quantification, a subset of them shed more than once, allowing for a measure of intershed interval. Younger toads from Experiment 1 had shorter intershed intervals (4.2 ± 0.63 days in 44 mm toads) than the older toads in Experiment 2 (6.3 ± 0.88 days in 103 mm toads). Another study also found that smaller cane toads shed more frequently than larger toads (Triana *et al.* 2013), possibly as a consequence of smaller toads undergoing rapid growth. There is evidence in other anurans that individuals with greater intershed intervals have skin bacteria that continuously grow over that entire time frame, resulting in greater bacterial loads before shedding than individuals with shorter intershed intervals (Cramp *et al.* 2014). Consequently, small toads that shed frequently may not reach similar bacterial density on their skin as older, larger toads. Though frequent shedding could limit the protective capacity of the skin bacteria, shedding can also keep pathogens including *Batrachochytrium dendrobatidis* at bay, particularly in hosts less susceptible to chytridiomycosis, the disease caused by this fungal pathogen (Ohmer *et al.* 2017). Adult cane toads have low susceptibility to chytridiomycosis (Brannelly *et al.* 2018), and regular shedding could play a role in the species’ defenses against infection.

Just as bacterial loads increased over time after shedding, so did multiple diversity metrics. Even though toads were housed in clean plastic containers with presumably minimal resources for reinoculation, we still found increasing diversity with time. Skin shedding, controlled by the corticotropin–corticosteroid hormone system in at least some Bufonids, is characterized by wiping behaviors stereotypic of the species to remove the sloughed skin, often followed by ingestion of the shed (Larsen 1976; Weldon *et al.* 1993; Castanho & de Luca 2001; Ohmer *et al.* 2015). These wiping behaviors may aid in bacterial retention, spreading microbes over the newly exposed skin. A previous study found that reductions in bacteria due to shedding are inconsistent between the dorsal and ventral sides of cane toads (Meyer *et al.* 2012). Therefore, the increase in diversity we found over time since shedding was likely influenced by bacterial spread from elsewhere on the body surface, rather than reinoculation from the environment. In the wild, increases in diversity are likely amplified compared with our lab‐based measurements. In green tree frogs, shedding frequently takes place at the beginning of the active part of the frogs’ day in the early evening (Cramp *et al.* 2014; Ohmer *et al.* 2015), providing wild individuals with immediate exposure to diverse environmental bacteria as they move about the landscape.

It is also important to note that the pre‐shed samples do not represent baseline values for each individual, as they were collected at varying times before the toad shed. Instead, comparing the pre‐ and post‐shed samples may provide a better indication of community stability within individual toads. In alpha diversity, that level of stability could be approximated based on whether pre‐shed values fell within the range of an individual's post‐shed values. A better indication of community stability within individuals may be found in the trajectories of community similarity in the context of beta diversity.

Our results suggest that bacterial communities change on a trajectory after shedding, which is particularly visible in principal coordinates space for some individuals that exhibit a linear course of community changes. Incorporating the pre‐shed sample illustrates the magnitude of change that occurs over time and due to shedding. Four of the seven toads showed a large shift between the first (pre‐shed) to the second (2–14 h post‐shedding) samples, with gradual changes in community structure toward becoming more similar to the initial samples over time. Community membership, however, shifted after shedding without returning to pre‐shed membership, possibly due to the lack of a bacterial reservoir in the toads’ housing.

Our results also showed patterns of community stability over short time frames. We found greater community stability (lower variability) between shed events versus a timeframe that included a shed. Other studies have reported that habitat can influence community stability, largely by affecting the presence of low‐abundance members; accordingly, in some frogs in the Ranidae family, more abundant microbes and lower diversity communities tend to be more stable (Harrison *et al.* 2019; Ellison *et al.* 2021). Unfortunately, our sample sizes were too small to assess correlations between variability and alpha diversity metrics. Regarding the impact of low‐abundant taxa on variability, unlike previous studies, focusing on the top 76 ASVs on the toads did not dramatically decrease variability on individuals (only 8% reduction) and did not impact the degree of influence of the shedding event on that variability. This could have been because the communities were in flux due to the relatively new environments. Had the toads in the present study been housed in their simple habitats for longer, we could expect their communities to become even more similar and stable over time.

Through this study, we found support for shedding in toads acting as a disruptor of skin communities, reducing bacterial loads and diversity. Shedding events are regular cyclical occurrences, keeping skin communities in a constant state of flux while retaining some relative stability within individuals over time. As a first glimpse into the changing community composition surrounding shedding events, many questions remain. For instance, measuring communities at more regular time intervals over multiple shedding events would help us understand short‐term community stability and trajectories of recolonization. Based on previous work (Cramp *et al.* 2014), we might expect that the degree of change in membership and structure over time could be due to the length of intershed intervals as well. However, it remains unknown whether longer intershed intervals result in continuous changes or periodic states of increased stability in diversity. More regular sampling of more individuals would determine whether there is a common pattern among, and within, individuals. Differences among individuals may alternatively result from shedding behaviors, which are predictable in some anurans but to our knowledge have not been thoroughly examined in cane toads. Identifying the influence of environmental reservoirs would also help clarify our results and place them into a larger context of wild organisms. Lastly, shedding can impact chytrid infections (Ohmer *et al.* 2017); understanding whether shedding plays a role in freeing up niche space for beneficial community members, or inhibits those beneficial members as is shown when bacteria are artificially removed (Becker & Harris 2010), could give us great insight into the ecology of this disease on susceptible and non‐susceptible frogs alike.

## CONFLICT OF INTEREST STATEMENT

None to declare.

## References

[inz212858-bib-0001] Apprill A , McNally S , Parsons R , Weber L (2015). Minor revision to V4 region SSU rRNA 806R gene primer greatly increases detection of SAR11 bacterioplankton. Aquatic Microbial Ecology 75, 129–137.

[inz212858-bib-0002] Bates D , Mächler M , Bolker B , Walker S (2015). Fitting linear mixed‐effects models using lme4. Journal of Statistical Software 67, 1–48.

[inz212858-bib-0003] Becker MH , Harris RN (2010). Cutaneous bacteria of the redback salamander prevent morbidity associated with a lethal disease. PLoS ONE 5, e10957. 20532032 10.1371/journal.pone.0010957 PMC2881031

[inz212858-bib-0004] Bolyen E , Rideout JR , Dillon MR *et al.* (2019). Reproducible, interactive, scalable and extensible microbiome data science using QIIME 2. Nature Biotechnology 37, 852–857. 10.1038/s41587-019-0209-9 PMC7015180 31341288

[inz212858-bib-0005] Brannelly LA , Martin G , Llewelyn J , Skerratt LF , Berger L (2018). Age‐and size‐dependent resistance to chytridiomycosis in the invasive cane toad *Rhinella marina* . Diseases of Aquatic Organisms 131, 107–120. 30460917 10.3354/dao03278

[inz212858-bib-0006] Callahan BJ , McMurdie PJ , Rosen MJ , Han AW , Johnson AJA , Holmes SP (2016). DADA2: High‐resolution sample inference from Illumina amplicon data. Nature Methods 13, 581–583. 27214047 10.1038/nmeth.3869 PMC4927377

[inz212858-bib-0007] Caporaso JG , Lauber CL , Walters WA *et al.* (2011). Global patterns of 16S rRNA diversity at a depth of millions of sequences per sample. PNAS 108, 4516–4522. 20534432 10.1073/pnas.1000080107 PMC3063599

[inz212858-bib-0008] Castanho LM , de Luca IMS (2001). Moulting behavior in leaf‐frogs of the genus *Phyllomedusa* (Anura: Hylidae). Zoologischer Anzeiger‐A Journal of Comparative Zoology 240, 3–6.

[inz212858-bib-0009] Christian K , Weitzman C , Rose A , Kaestli M , Gibb K (2018). Ecological patterns in the skin microbiota of frogs from tropical Australia. Ecology and Evolution 8, 10510–10519. 30464823 10.1002/ece3.4518 PMC6238143

[inz212858-bib-0010] Conlon JM (2011). The contribution of skin antimicrobial peptides to the system of innate immunity in anurans. Cell and Tissue Research 343, 201–212. 20640445 10.1007/s00441-010-1014-4

[inz212858-bib-0011] Cramp RL , McPhee RK , Meyer EA , Ohmer ME , Franklin CE (2014). First line of defence: The role of sloughing in the regulation of cutaneous microbes in frogs. Conservation Physiology 2, cou012. 27293633 10.1093/conphys/cou012 PMC4806747

[inz212858-bib-0012] Ellison S , Knapp R , Vredenburg V (2021). Longitudinal patterns in the skin microbiome of wild, individually marked frogs from the Sierra Nevada, California. ISME Communications 1, 45. 37938625 10.1038/s43705-021-00047-7 PMC9723788

[inz212858-bib-0013] Fushida A , Riedel J , Nordberg EJ , Pillai R , Schwarzkopf L (2020). Can geckos increase shedding rate to remove fouling? Herpetologica 76, 22–26.

[inz212858-bib-0014] Greenspan SE , Migliorini GH , Lyra ML *et al.* (2020). Warming drives ecological community changes linked to host‐associated microbiome dysbiosis. Nature Climate Change 10, 1057–1061.

[inz212858-bib-0015] Harrison XA , Price SJ , Hopkins K , Leung WT , Sergeant C , Garner TW (2019). Diversity‐stability dynamics of the amphibian skin microbiome and susceptibility to a lethal viral pathogen. Frontiers in Microbiology 10, 2883. 31956320 10.3389/fmicb.2019.02883 PMC6951417

[inz212858-bib-0016] Harris‐Tryon TA , Grice EA (2022). Microbiota and maintenance of skin barrier function. Science 376, 940–945. 35617415 10.1126/science.abo0693

[inz212858-bib-0017] Jiménez RR , Sommer S (2017). The amphibian microbiome: Natural range of variation, pathogenic dysbiosis, and role in conservation. Biodiversity and Conservation 26, 763–786.

[inz212858-bib-0018] Larsen LO (1976). Physiology of moulting. In: Lofts B , ed. Physiology of the Amphibia. Academic Press, New York, pp. 53–100.

[inz212858-bib-0019] Meyer EA , Cramp RL , Bernal MH , Franklin CE (2012). Changes in cutaneous microbial abundance with sloughing: Possible implications for infection and disease in amphibians. Diseases of Aquatic Organisms 101, 235–242. 23324420 10.3354/dao02523

[inz212858-bib-0020] Ohmer ME , Cramp RL , White CR , Franklin CE (2015). Skin sloughing rate increases with chytrid fungus infection load in a susceptible amphibian. Functional Ecology 29, 674–682.

[inz212858-bib-0021] Ohmer MEB , Cramp RL , Russo CJM , White CR , Franklin CE (2017). Skin sloughing in susceptible and resistant amphibians regulates infection with a fungal pathogen. Scientific Reports 7, 3529. 28615642 10.1038/s41598-017-03605-z PMC5471217

[inz212858-bib-0022] Ohmer MEB , Cramp RL , White CR *et al.* (2019). Phylogenetic investigation of skin sloughing rates in frogs: Relationships with skin characteristics and disease‐driven declines. Proceedings of the Royal Society B: Biological Sciences 286, 20182378. 10.1098/rspb.2018.2378 PMC6408608 30963925

[inz212858-bib-0023] Oksanen J , Blanchet FG , Friendly M *et al.* (2020). vegan: community ecology package, R Package Version 2.5–7. Available from URL: https://CRAN.R‐project.org/package=vegan

[inz212858-bib-0024] Parada AE , Needham DM , Fuhrman JA (2016). Every base matters: Assessing small subunit rRNA primers for marine microbiomes with mock communities, time series and global field samples. Environmental Microbiology 18, 1403–1414. 26271760 10.1111/1462-2920.13023

[inz212858-bib-0025] Quast C , Pruesse E , Yilmaz P *et al.* (2012). The SILVA ribosomal RNA gene database project: Improved data processing and web‐based tools. Nucleic Acids Research 41, D590–D596. 23193283 10.1093/nar/gks1219 PMC3531112

[inz212858-bib-0026] R Core Team (2022). R: A Language and Environment for Statistical Computing. Available from URL: https://www.r‐project.org/

[inz212858-bib-0027] RStudio Team (2020). RStudio: Integrated Development Environment for R. RStudio, PBC, Boston, MA.

[inz212858-bib-0028] Russo CJ , Ohmer ME , Cramp RL , Franklin CE (2018). A pathogenic skin fungus and sloughing exacerbate cutaneous water loss in amphibians. Journal of Experimental Biology 221, jeb167445. 29752415 10.1242/jeb.167445

[inz212858-bib-0029] Stabler RM (1939). Frequency of skin shedding in snakes. Copeia 1939, 227–229.

[inz212858-bib-0030] Triana TM , Henao LM , Bernal MH (2013). Comparación ontogénica de la frecuencia de muda en *Rhinella marina* (Anura, Bufonidae). Iheringia. Série Zoologia 103, 47–50.

[inz212858-bib-0031] Voyles J , Rosenblum EB , Berger L (2011). Interactions between *Batrachochytrium dendrobatidis* and its amphibian hosts: A review of pathogenesis and immunity. Microbes and Infection 13, 25–32. 20951224 10.1016/j.micinf.2010.09.015

[inz212858-bib-0032] Weldon PJ , Demeter BJ , Rosscoe R (1993). A survey of shed skin‐eating (dermatophagy) in amphibians and reptiles. Journal of Herpetology 27, 219–228.

[inz212858-bib-0033] Woodhams DC , McCartney J , Walke JB , Whetstone R (2023). The adaptive microbiome hypothesis and immune interactions in amphibian mucus. Developmental & Comparative Immunology 145, 104690. 37001710 10.1016/j.dci.2023.104690 PMC10249470

[inz212858-bib-0034] Wu NC , Cramp RL , Franklin CE (2017). Living with a leaky skin: Upregulation of ion transport proteins during sloughing. Journal of Experimental Biology 220, 2026–2035. 28566357 10.1242/jeb.151738

[inz212858-bib-0035] Wu NC , McKercher C , Cramp RL , Franklin CE (2019). Mechanistic basis for loss of water balance in green tree frogs infected with a fungal pathogen. American Journal of Physiology‐Regulatory, Integrative and Comparative Physiology 317, R301–R311. 31141416 10.1152/ajpregu.00355.2018

[inz212858-bib-0036] Yilmaz P , Parfrey LW , Yarza P *et al.* (2014). The SILVA and “all‐species living tree project (LTP)” taxonomic frameworks. Nucleic Acids Research 42, D643–D648. 24293649 10.1093/nar/gkt1209 PMC3965112

